# Event-based high-resolution neutron image formation analysis using intensified CMOS cameras

**DOI:** 10.1038/s41598-024-78104-z

**Published:** 2024-11-06

**Authors:** Alex Gustschin, Yiyong Han, Adrian Losko, Alexander Wolfertz, Daniel S. Hussey, László Szentmiklósi, Zoltán Kis, Pavel Trtik, Pierre Boillat, Anders Kaestner, Markus Strobl, Alessandro Tengattini, Lukas Helfen, Michael Schulz

**Affiliations:** 1 Technical University of Munich, Heinz Maier-Leibnitz Zentrum (MLZ), Lichtenbergstr. 1, 85748 Garching, Germany; 2 National Institute of Standards and Technology (NIST), Gaithersburg, MD, 20899-8461 USA; 3 Nuclear Analysis and Radiography Department, Centre for Energy Research, Budapest, Hungary; 4 Laboratory for Neutron Scattering and Imaging, Paul Scherrer Institut (PSI), 5232 Villigen, Switzerland; 5 Institut Laue-Langevin (ILL), 38042 Grenoble Cedex 9, France; 6 Univ. Grenoble Alpes, Grenoble INP, CNRS, 3SR, 38000 Grenoble, France

**Keywords:** Imaging techniques, Imaging and sensing

## Abstract

We present a versatile optical setup for high-resolution neutron imaging with an adaptable field of view and magnification that can resolve individual neutron absorption events with an image intensifier and a CMOS camera. Its imaging performance is characterized by evaluating the resolution limits of the individual optical components and resulting design aspects are discussed. Neutron radiography measurements of a Siemens star pattern were performed in event mode acquisition comparing two common high-resolution neutron scintillators, crystalline Gadolinium Gallium Garnet (GGG) and powdered Gadolinium Oxysulfide (GOS). An analysis of the light signature caused by neutron absorption events is performed and some resulting issues for both GGG and GOS regarding optical system design are addressed. Both scintillators reach similar resolution (4–5 ${\upmu }$m) in event mode acquisition despite different light emission characteristics. The findings suggest that, in the case of GOS, the resolution is limited by the size of the light clusters which in turn originate from the photon scattering at the boundaries of the powder particles comprising it, while with GGG the lower light conversion efficiency makes it challenging to collect enough photons to trigger sufficient signal amplification in the image intensifier. Overall, the proposed event-based evaluation of scintillators allows for quantifying and optimizing various design parameters, which is much more complex than adopting conventional methods based on integrated images.

## Introduction

High-resolution imaging has been constantly developed for various types of radiation and has played a vital role in addressing countless scientific questions. While optical-, X-ray-, electron- and several types of scanning probe microscopy have already reached the nanometre range, neutron radiography is limited to several $\mu$m resolution. Nevertheless, the demand for higher resolutions has increased in recent years, as advanced neutron imaging applications such as the study of water transport in fuel cells^1,2^, dendrite formation and lithium distribution dynamics in batteries^3,4^ and other unique science cases have emerged^5–7^. One of the challenges in high-resolution neutron imaging is the comparatively low flux and strong collimation requirements typical of neutron sources. In addition, high-energy secondary particles are involved in the image formation process, causing a strongly blurred light emission in the scintillator material. Several other imaging techniques that also rely on high-energy radiation such as Positron Emission Tomography face similar challenges in terms of resolution, since physical interactions have to be reconstructed from highly statistical processes. In the case of neutron radiography, a suitable material that absorbs neutrons and transforms them into light is usually used. To achieve high spatial resolution, thin layers of such materials—denoted as scintillator screens—are coupled to magnifying optics to generate the transmission image, which is projected onto the image sensor. When the converter in the scintillator absorbs neutrons via nuclear reactions, various high-energy particles are ejected. These can further interact with the scintillator material or leave the latter without further energy transfer and light generation. While lithium fluoride (LiF) with zinc sulfide (ZnS) additives is the most common scintillator due to its high light conversion efficiency, its absorption efficiency for neutrons is very low in the sub-50 ${\upmu }$m thickness range^8^. Hence high-resolution neutron imaging, which requires thin scintillators, is mostly performed with scintillators based on gadolinium (Gd). They provide an almost ideal absorption performance but suffer from low light conversion and hence are limited by photon noise. Recently, borated ZnS:Cu screens were also evaluated for high-resolution applications and showed a significantly higher light conversion than comparable Gd-based screens^9^. However, in the sub-10 ${\upmu }$m resolution range, Gd as converter material is the most common option.

In Gd-based scintillators, $\gamma$-rays in the MeV range and high energy electrons (29.3–926.8 keV) are emitted via the $^{157}$Gd(n, $\gamma$) neutron capture reaction. Since the other isotopes of Gd have a much smaller absorption cross section with neutrons, their contribution to the signal formation is minor. While the prompt gamma rays hardly interact with the scintillator due to their high energy, the internal conversion electrons (ICE) with the lower energies are slowed down ionizing the surrounding material and causing scintillation processes with an isotropic light emission. The vacancies created after the ejection of the ICEs are rapidly filled up, resulting in further emission of fluorescent X-rays or Auger electrons. According to^10^ the most abundant and relevant electron energies are 29.3 keV, 71.7 keV, and 34.9 keV Auger electrons, whereas the higher energies (>100 keV) hardly play a role in light generation due to their lower output and poor self-absorption probabilities of thin Gd-based scintillators. Some works simulated and measured the conversion efficiency of thin Gd foils coupled to silicon semiconductor detectors which allows measuring the energy of the ICEs^10,11^. The films have to be several $\upmu$m thick to absorb the neutrons but still allow the ICEs to exit the Gd foil to be detected. In Gd scintillators, however, the ICEs should deposit most of their energy in the phosphor layer for high light efficiency. While a higher scintillator thickness increases the overall neutron absorption as well as the overall emitted light by the electrons, it also leads to scattering and unwanted background illumination which decreases the resolution. Therefore - having to find a trade-off between light conversion efficiency and spatial scintillator blur—a quantitative method to measure the response of the scintillator to single neutron detection events is of great benefit.

In recent years, several high-resolution neutron radiography systems have been developed and investigated^12–17^. They mostly rely on Gd as conversion material and either use crystalline gadolinium gallium garnet $\textrm{Gd}_3\textrm{Ga}_5\textrm{O}_{12}$ (further referred to as GGG)^17^, gadolinium aluminum gallium garnet $\textrm{Gd}_3\textrm{Al}_2\textrm{Ga}_3\textrm{O}_{12}$ (GAGG)^16^ or powder-based $\textrm{Gd}_2\textrm{O}_2\textrm{S}$ (GOS) phosphor screens^12–15^. With the latter approach the GOS powder has been prepared with a high fraction of $^{157}$Gd to increase neutron absorption and the thickness has been reduced down to 3.5 ${\upmu }$m to reach a resolution of $\approx$ 5 ${\upmu }$m^14^. At such thin layers, the fraction of ICEs escaping the scintillator without having deposited most of their energy is relatively high—especially if the neutrons are absorbed close to the surface of the phosphor layer. In a related work^18^ the scintillator material was deposited on iridium layers on the substrate to exploit the backscattered electrons and reflected photons for a higher light yield. The disadvantage of such screens is their grainy structure which is superimposed on the image and is to some extent corrected with a reference image without the sample in the beam. However, refractive perturbations hardly allow to increase the resolution beyond the size range of the phosphor particles from a single exposure even with extremely thin layers. Thin crystalline scintillators today widely used in high-resolution X-ray detectors do not have that problem and usually have a much more homogenous illumination^17^. They even allow focusing on different planes inside the transparent crystal. This concept was realized for neutron imaging with a 20 ${\upmu }$m thick Eu-doped GGG scintillator by Tengattini et al.^17^ reaching sub-4 ${\upmu }$m resolution. It was found to be 35% less light efficient compared to a 3 ${\upmu }$m GOS scintillator with enriched $^{157}$Gd. One aspect that limits the light efficiency of this type of scintillator is total reflection from the crystal surface. It hinders a high fraction of the light from exiting the scintillator towards the lens and effectively reduces its numerical aperture.

Yet another approach is to amplify the light signal in the optical system by using image intensifiers. Such a device consists of a microchannel plate (MCP) that multiplies photon-induced electrons which then generate an intensified image on a phosphor screen. These devices also reduce the resolution due to the finite size of the microchannels, but they can amplify signals generated by single neutron absorption events to a degree that they can be easily recorded by image sensors. This led to the development of event-based acquisition schemes which were shown to improve imaging performance^15,19–21^. Similar to photon-counting detectors used e.g. in x-ray applications, event-based neutron detectors are designed to record the signature of individual neutron absorption processes in the scintillator material. Several challenges arise with this acquisition approach: the frame rate or readout time of the image sensor has to be fast enough to cope with the neutron flux. The emitted light has to be amplified to create a signal surpassing the electron noise of the image sensor. Finally, an algorithm must identify and spatially localize the neutron absorption effect filtering out parasitic signals like scattered photons, gamma-events, or dark events of the image intensifier. After processing the neutron events, the image can be reconstructed with a user-defined pixel size and therefore enables sub-pixel resolution^15,19–21^. Other advantages include the reduction of noise and the possibility of performing energy-resolved imaging when using pulsed neutron sources by time-of-flight measurements^20,21^. The latter, however, requires event-based imaging sensors, that can temporally resolve the cascade of emitted photons from a distinct event. Unlike the here presented setup with a CMOS camera with exposure times in the range of 5–10 ms, event-based pixel sensors can use the time signature of the detected photons attributed to a neutron absorption event to minimize the influence of the scintillator afterglow^21^.

In this work, we developed a compact, modular optical system with an adaptable field of view and magnification suitable for high-resolution neutron imaging in both conventional integrated and event modes. The system is optically characterized in terms of resolution with and without an image intensifier to evaluate the respective limitations. Centroiding algorithms were applied to determine the center of mass (CoM) positions of individual, amplified events stemming from the scintillator screen. High-resolution neutron radiographs were performed with both a GGG and GOS scintillator in event mode. Using the acquired data the image formation process is further investigated and possibilities for scintillator characterization with the proposed setup are discussed.

## Methods

### Experimental setup

**Fig. 1:**
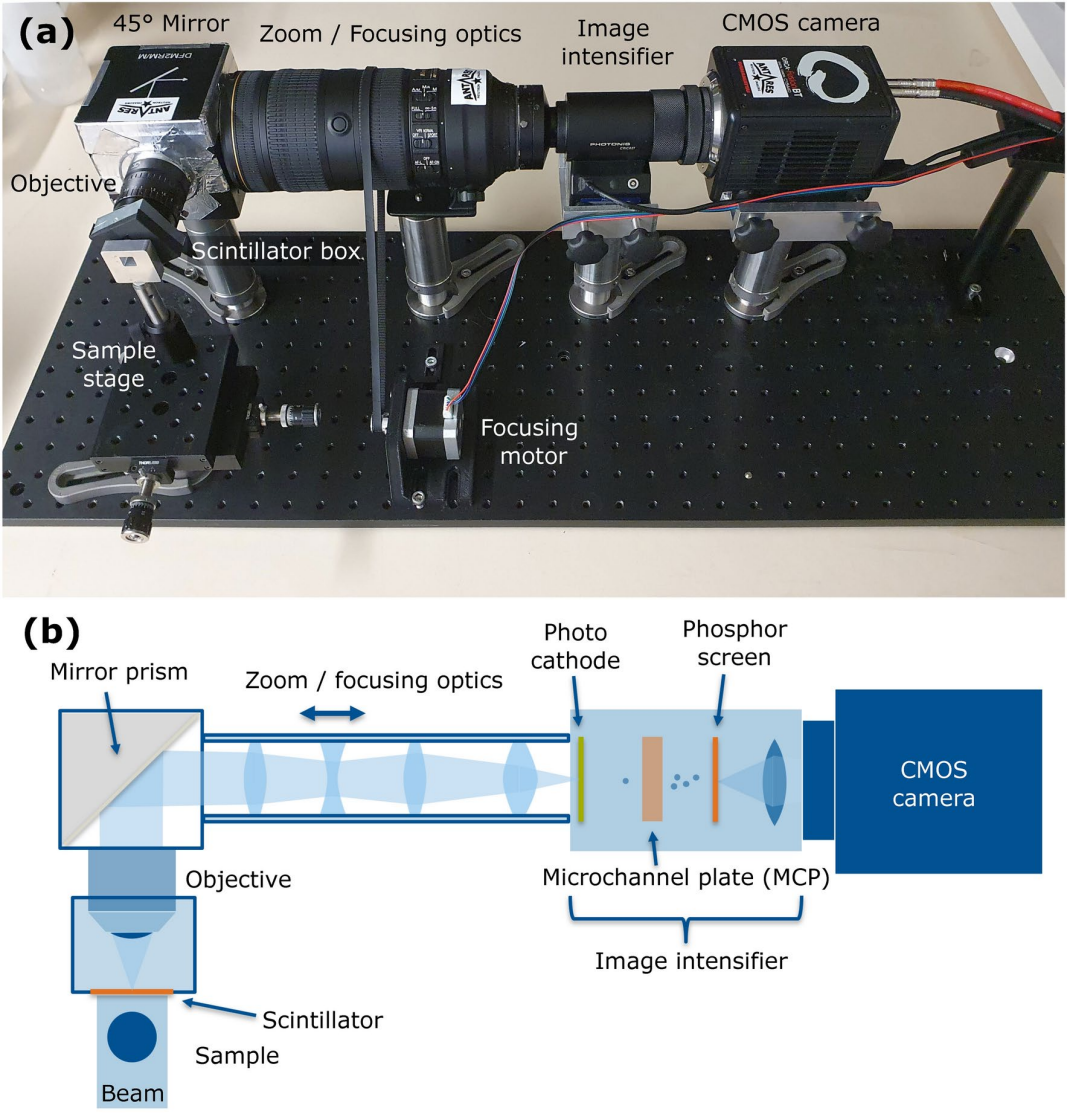
Photograph (**a**) of the neutron imaging setup with labeling of individual components and (**b**) schematic illustration of the beam path through the magnifying optics and image intensification.

The imaging setup is shown in Fig. 1a with a schematic illustration of the beam path (b) through the magnifying optics and the image intensifier. It is a compact, modular assembly composed of mostly off-the-shelf components, and provides high flexibility for straightforward modification with other optical components and cameras allowing users to tailor its functionality to their specific preferences. The scintillator is fixed on a custom-designed C-mount holder which is screwed onto a NAVITAR 25 mm (DO-2595) objective lens. The latter is attached to a 60 mm beam-turning cage with a $45^{\circ }$ mirror prism (Thorlabs DFM2RM/M) to divert the optical path out of the neutron beam and protect the sensitive parts from ionizing radiation. A Nikon AF-S NIKKOR 70–200 mm zoom lens is coupled to the other part of the mirror box and allows for zooming and focusing the scintillation plane. The focusing is implemented by a stepper motor which is strapped to the focussing mechanism. The system allows total optical magnifications of 3–8$\times$ (continuously adjustable) and effective pixel sizes of 0.8–2.2 ${\upmu }$m. The field of view (FoV) that is achieved with the system ranges from 1.8 to 4.2 mm in diameter. The image intensifier (Cricket PP3050G, Photonis Scientific Detectors) is placed between the ocular lens system and the CMOS camera. It has a quantum efficiency of $\approx 0.15$ (545 nm—main GOS emission wavelength) and amplifies the signal by a factor of ${10}^4$ according to manufacturer specifications. Its resolution is specified at 60lp/mm and is close to the Nyquist limit of the image sensor pixel size. The camera is a high-speed Hamamatsu ORCA-Fusion BT C15440 with a pixel size of 6.5 ${\upmu }$m on an array of $2304 \times 2304$ pixels. In the event mode acquisition, it was operated at 5 ms exposure time (200Hz) with a region of interest (ROI) of $800 \times 800$ pixels (16bit) with a typical data rate of 270MB/s. This frame rate is compatible with the decay time of the used scintillators (GOS:Tb 1.5 ms and GGG:Eu 0.8 ms).

**Fig. 2:**
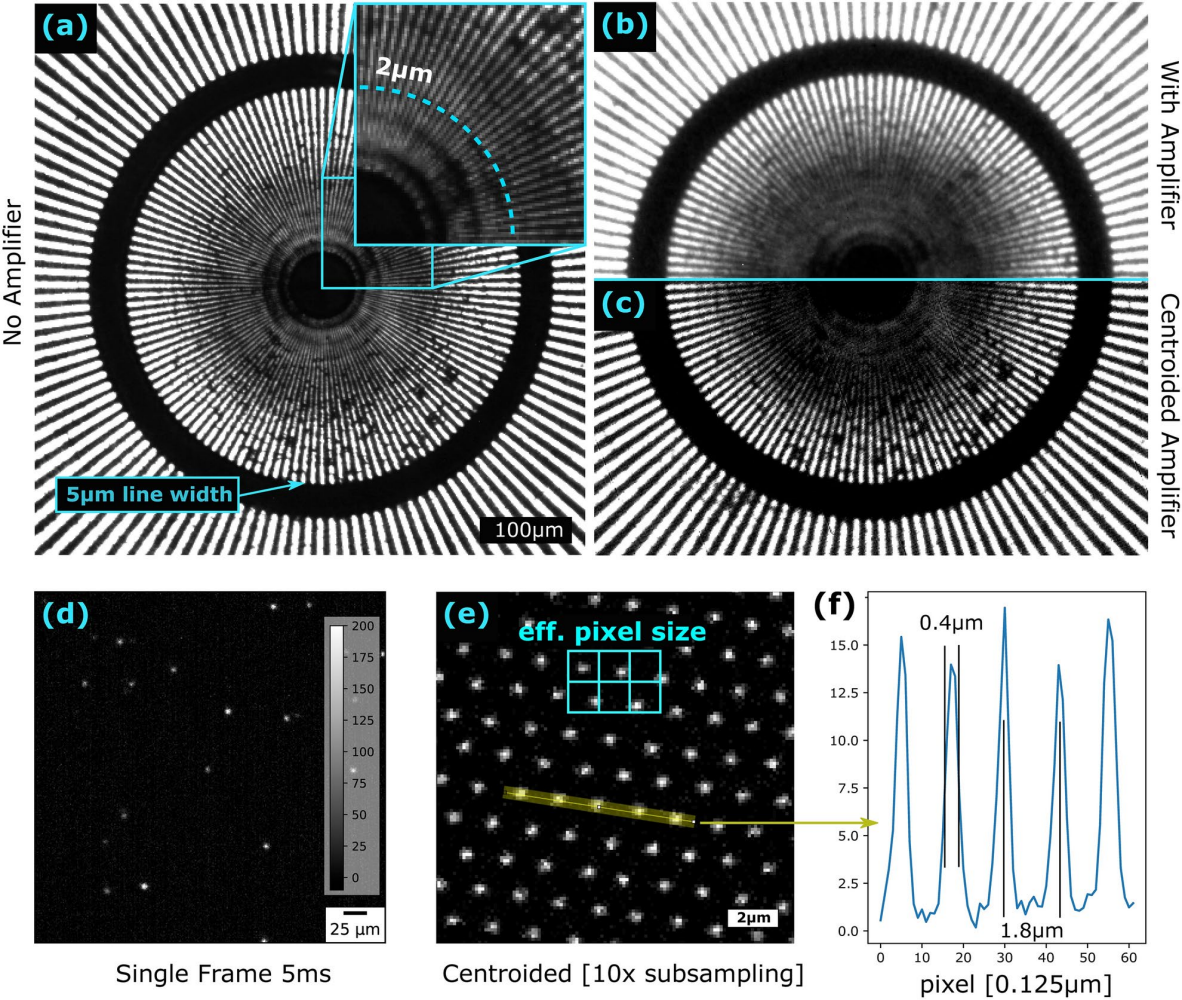
Optical characterization of the imaging system. (**a**) Integrated image without image intensifier, (**b**) integrated image with image intensifier, (**c**) acquisition with image intensifier in event-mode and subsequent reconstruction with 1.25 ${\upmu }$m pixel size. (**d**) Representative frame of event acquisition with 5 ms exposure after dark current correction. (**e**) Reconstruction of the optical event measurement with $10\times$ subsampling with a virtual pixel size of 0.125 ${\mu }$m and comparison to the effective pixel size (1.25 ${\upmu }$m after optical magnification). (**f**) Line plot along the hexagonal array showing the optically magnified period of the MCP of 1.8 ${\upmu }$m.

### Optical characterization

To characterize the optical resolution, we used the same Gd-based Siemens star test pattern (acquired from Paul-Scherrer Institute, Switzerland) later used for neutron radiography. It consists of Gadolinium structured on a transparent glass substrate, which allows for high contrast in both visible light and neutron imaging and enables a direct comparison with the neutron radiography data. As a light source, a low-intensity green LED (<1 mm spot of light emission) was placed 2.4 m from the resolution pattern to provide a quasi-parallel light beam. A diaphragm was placed 0.6 m before the resolution pattern to control the intensity by changing the aperture size. The beam path after the diaphragm was optically isolated to prevent the influence of stray light. For the tests, the zoom level was adapted for an effective pixel size of 1.25 ${\upmu }$m and an optical magnification of $\times 5.2$. First, the system was tested without the image intensifier. It was manually focused and 10 frames with an exposure time of 1 s were acquired and averaged. The image without the amplifier is given in Fig. 2 (a) with a magnified inset, showing that the resolution is essentially limited by the pixel size. Afterward, the image intensifier was introduced into the system and was operated at maximum amplification. To avoid overexposure, the aperture before the sample was strongly reduced till the measured intensities were compatible with the dynamic range of the camera. After optimizing the focus on both the zoom lens and the intensifier, 60 frames with 1 s exposure time were taken. In the following part, the system was tested in event mode, i.e. the exposure time was set to 5 ms, and the intensity was reduced to a degree where isolated spots representing single photons were well distinguishable on a single frame. ${2.4}\times {10}^5$ frames (20 min) were recorded for the reconstruction with centroiding algorithms^15^. Note that the image acquisition in the different modes required significantly different total exposure times to cope with hardware-related limitations. In this case, where the optical resolution and the influence of the image intensifier were tested, the exposure time was chosen long enough to provide sufficient photon statistics. Figure 2 shows a comparison of the image taken with the intensifier in integrated (b) and event-reconstructed mode by centroiding (c). Note that this image is not normalized by a respective scan without the sample, since the Siemens star pattern was mounted instead of the scintillator. Hence, some artifacts related to the structures in the MCP are visible.

### High-resolution neutron radiography

The system was tested for neutron radiography at the NIPS-NORMA station^22^ at the Budapest Neutron Center (BNC). To achieve high resolution in the sub-10 ${\upmu }$m range the pinhole was chosen to be 1$\times {1}\,\textrm{cm}$ which resulted in an *L*/*D* ratio of 500. At the beginning of each measurement, dark current frames were acquired and the scintillator screen was focused thanks to several integrated exposures. To determine the resolution, a Gd-based Siemens star (acquired from Paul Scherrer Institute, PSI) was placed as close as possible to the screen, but so that it could still be moved to the side by a motorized stage for the “open beam” reference acquisition. The sample distance from the scintillating plane is estimated to be < 1 mm which would suggest a geometrical blur of < 2 ${\upmu }$m at the given *L*/*D* ratio. Neutron radiographs were acquired with two scintillators: (1) powder-based GOS:Tb of 5 ${\upmu }$m thickness with enriched $^{157}$Gd content acquired from PSI and (2) a crystalline gadolinium gallium garnet (GGG:Eu) with a 20 ${\upmu }$m thick layer of Eu doping (provided by ESRF, Grenoble). The optical magnification was adjusted to $\times 5.9$ which resulted in an effective pixel size of 1.1 ${\upmu }$m. The exposure time per frame was 5 ms and allowed continuous acquisition at an ROI of $800 \times 800$ pixels. The total acquisition time was 6 h for open beam and sample exposure each resulting in ${4.32\times {10}^6}$ single frames. Note that the long exposure time results from extremely narrow beam collimation and rather non-optimized beamline conditions for imaging in terms of flux. Additionally, event measurements were undertaken with a 10 ${\upmu }$m thick GOS scintillator (RC TRITEC, Switzerland), which is not isotopically enriched like the 5 ${\upmu }$m GOS. The acquisition parameters were the same to compare the generated light clusters with the thinner GOS 5 ${\upmu }$mm scintillator.

**Fig. 3:**
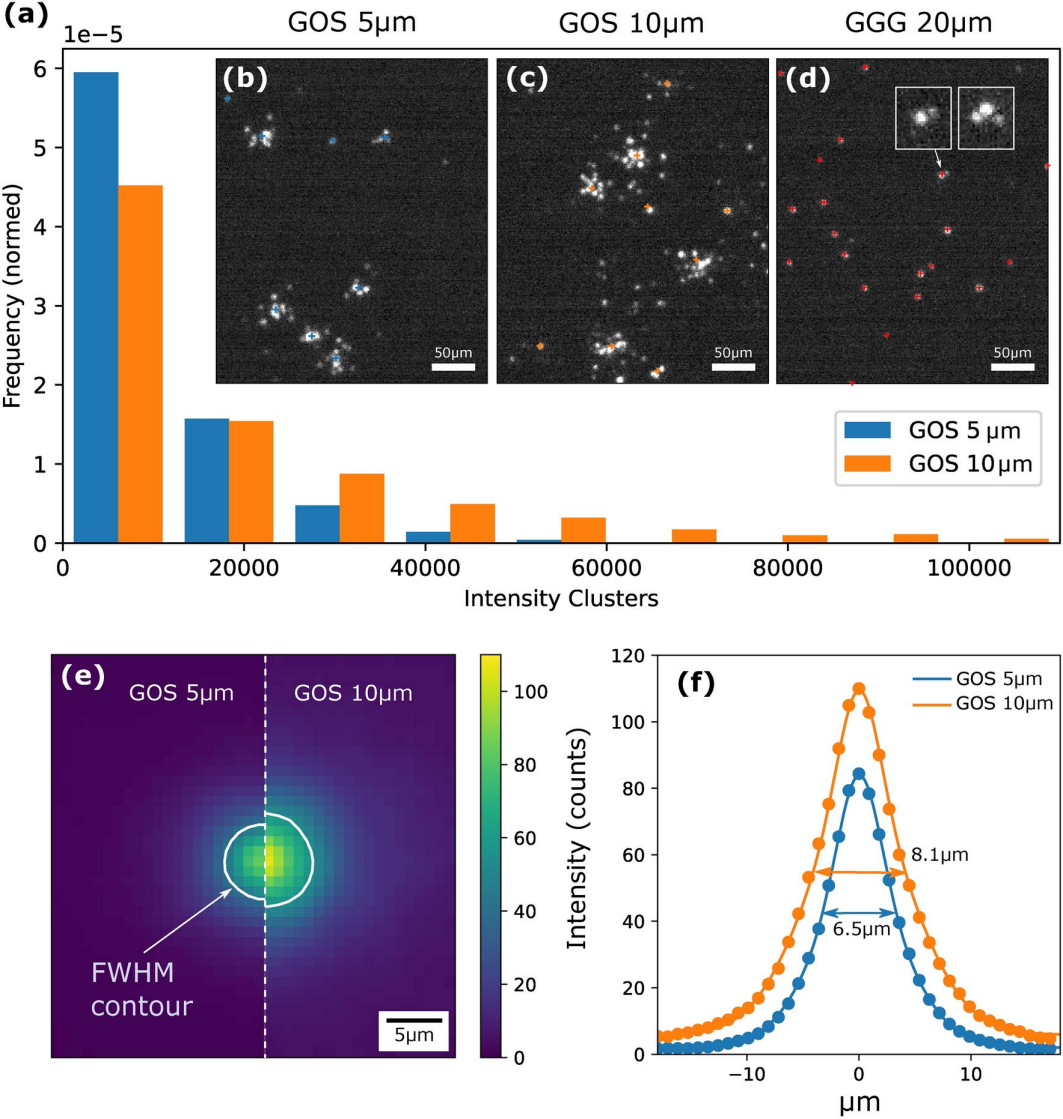
(**a**) Histogram of the cluster intensities with both GOS scintillators. Representative frames of short exposures showing the light signature of neutron impact on the scintillators GOS 5 ${\upmu }$m (**b**) and GOS 10 ${\upmu }$m (**c**) with respective marks showing the determined centers of the clusters. (**d**) A representative frame of the GGG 20 ${\upmu }$m exposure shows mainly isolated dots with some rarely-occurring cluster-like structures of 2 or 3 close intensity spots (magnified insets). The light spots from the GGG scintillator are not included in the histogram comparison since they are all of comparable size and do not show the characteristics of clusters. (**e**) Comparison of many clusters averaged showing the estimated light spread distribution generated by a neutron absorption event for both GOS scintillators with a contour at the full width at half maximum (FWHM). (**f**) Line plots with double-Gaussian fit along the dashed line in (**e**) for both distributions with respective values for the FWHM.

### Data processing

The centroiding and image reconstruction algorithm is based on a procedure described previously by Hussey et al.^15^ In the first step, the averaged dark image was subtracted from each frame. After that, a threshold filter was applied to set the noise background to zero. Then, the identification of contiguous pixels with intensity above the threshold is conducted by identifying the intensity spots. For each spot, the center of mass (CoM) in the x- and y-directions as well as its total intensity and number of comprising pixels, is calculated and saved. The image reconstruction is carried out by defining a quadratic grid of virtual pixels (which can be smaller than the actual pixel size for sub-pixel resolution) and the assignment of the individual events as counts in the corresponding pixels based on their centroided coordinates. The choice of the pixel size for reconstruction depends on several aspects. Firstly, a trade-off between Poisson noise and the pixel size (limiting the resolution) needs to be made. A subsampling by factor 2 (halving the pixel size) requires increasing the exposure time by a factor of 4 to reach comparable noise levels. Secondly, using an image intensifier with a regular MCP matrix (typically hexagonal) introduces Moiré artifacts with the pixel array of the camera if their periods are similar. With an even stronger subsampling the hexagonal structure of the image intensifier’s MCP can be resolved as shown in Fig. 2e ($10\times$ subsampling, 0.125 ${\upmu }$m virtual pixel size) with a comparison to the real, optically magnified pixel size. However, such a high subsampling beyond the period of the MCP is not helpful for imaging performance since the pixels with low intensity will produce a noisy signal after normalization with the corresponding reference image. A line plot (Fig. 2f) shows an effective period of 1.8 ${\upmu }$m of the MCP matrix, which can be considered as the physical resolution limit for optical performance.

An event analysis of the neutron radiography data was carried out to compare the light clusters generated by the three scintillators. Figure 3b–d shows exemplary frames acquired at 5ms exposure after dark image correction. While the GGG (d) produces mainly single spots similar to the ones in the optical tests, the powder scintillators GOS 5 ${\upmu }$m and GOS 10 ${\upmu }$m produce larger, distinct light clusters that have several related spots that can be as far as 15 ${\upmu }$m away from the cluster centers. For quantitative analysis, we used an intensity-weighted K-means clustering algorithm and processed the first 100 frames for the GOS 5 ${\upmu }$m and GOS 10 ${\upmu }$m scintillators. A comparison of cluster intensity (sum of pixel counts associated with the cluster) is given by the normed histogram in Fig. 3a. After cluster identification, the clusters were isolated ($40\times 40$-pixel window around their CoMs) and saved in a separate array. Clusters with lower than 3000 counts of total intensity were excluded since they usually consisted of one single isolated spot that is not typically ascribed to a neutron absorption event but rather to scattered single photons, scintillator afterglow, or dark current from the intensifier. The remaining cluster arrays were averaged, yielding a statistical intensity distribution expected from a neutron absorption event. To some extent this can be understood as an estimate of the point spread function (PSF), however, one has to consider that the absorption point of the neutron does not necessarily coincide with the point where light generation starts by the secondary particles. These averaged intensity distributions are shown in Fig. 3e to compare the GOS 5 ${\upmu }$m and GOS 10 ${\upmu }$m screens with the contour at FWHM. Fig. 3f shows line plots along the dashed, vertical line in (e).

**Fig. 4:**
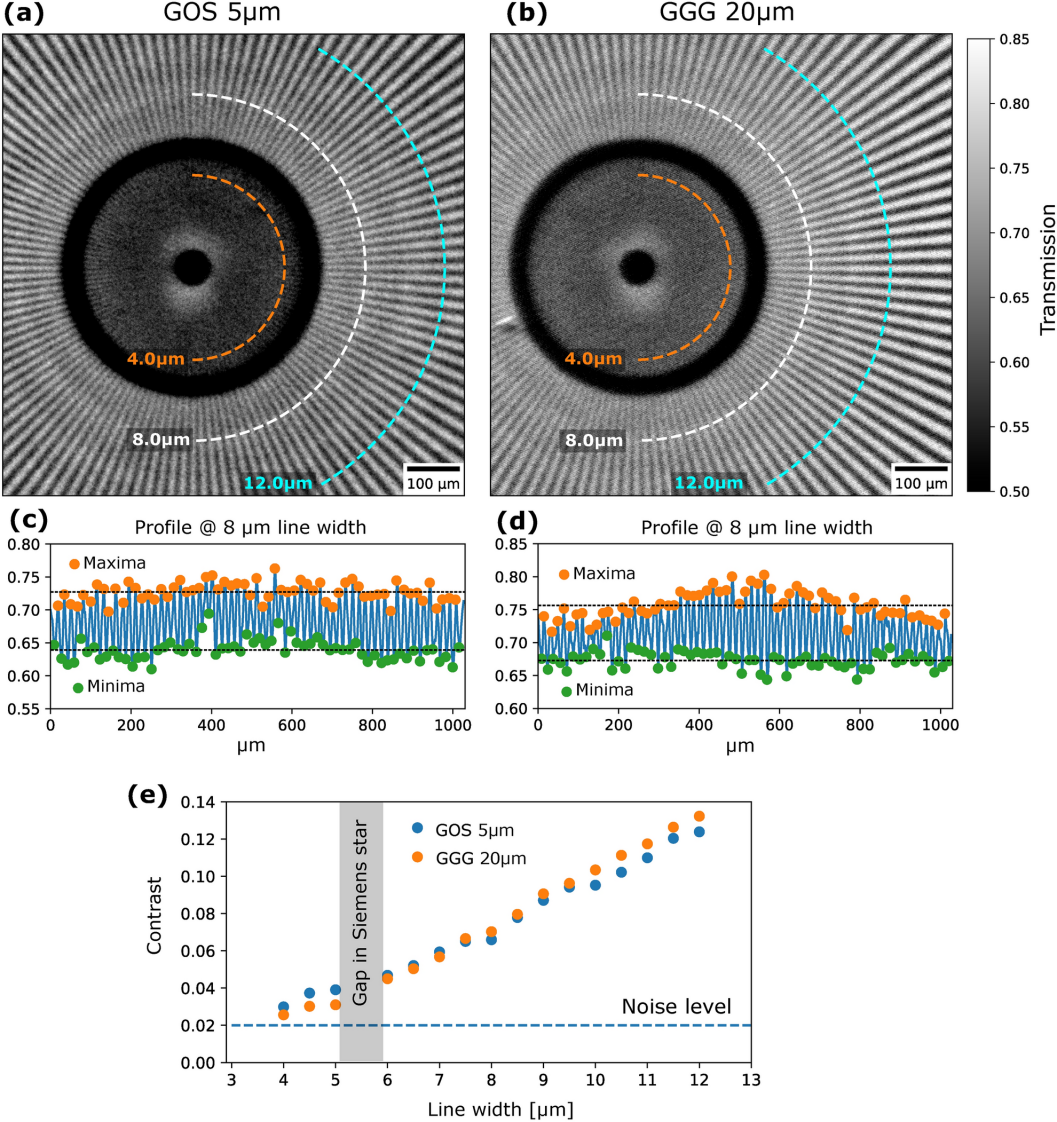
Reconstructed neutron radiographs of the Siemens start pattern for (**a**) GOS 5 ${\upmu }$m scintillator and (**b**) the GGG 20 ${\upmu }$m scintillator. Representative radial line profiles at 8 ${\upmu }$m line width for GOS 5 ${\upmu }$m (**c**) and GGG 20 ${\upmu }$m (**d**) with mean values for maxima and minima of intensity used for the contrast calculation. (**e**) Contrast at increasing line width from 4 to 12 ${\upmu }$m along the arcs indicated in the respective radiographs (**a**) and (**b**).

The image reconstruction of the neutron radiographs was performed with the same procedure as in the optical characterization case. For long exposures such as the ones in the present study, a sample drift (*e.g.*, due to thermal fluctuations, mechanical relaxations, vibrations, etc) also has to be considered. The movement of the sample by 1 ${\upmu }$m over 6 h would already critically impact the image in the aimed resolution range. In this case, the sample drift was corrected by calculating consecutive temporal images and determining the displacement to the previous frame through digital image correlation. This was carried out in several steps. First, a Gaussian filter with a kernel of 5 pixels was applied for noise reduction. After that, the images were digitally shifted in subpixel steps (0.2 pixels) in x- and y-directions, and the difference to the previous image (residuum) was calculated. The shift in the x- and y-direction with the lowest residuum was considered the most reliable displacement. After that, the displacement over all images in the x- and y-direction was separately fitted with a linear equation, and from that the displacement of every intermediate image was calculated. In the last step, the images were averaged after respective spatial drift correction. Additional information elaborating on the effect of the drift correction is given in Fig. S1 in the supplementary information. It should be noted that the setup was originally designed for the instrument ANTARES at FRM II^23^, which provides a temperature-controlled and more stable measurement environment, and such corrections might not be necessary in the future depending on the facility adopted. Figures 4a and b show the acquired radiographs with the GOS 5 ${\upmu }$m and the GGG 20 ${\upmu }$m scintillators. The resolution was determined by half-circular line plots around the center of the Siemens star averaged over 5 pixels, denoted in Fig. 4a and b by the dashed lines. The line plots were first filtered with a Savitzky-Golay-Filter (5-pixel window size) to further reduce the influence of noise and better identify the periodic maxima and minima. Resulting profiles for a line width of 8 ${\upmu }$m (period of the lines 16 ${\upmu }$m) are shown in Fig. 4c for the GOS 5 ${\upmu }$m and in Fig. 4d for the GGG. The average maxima and minima (denoted by the dashed lines) were calculated for each radial profile and the contrast for each line width was determined by the formula $C = (I_{max}-I_{min}) /(I_{max}+I_{min})$. This procedure was performed on arcs from 4.0 to 12 ${\upmu }$m line width and the respective contrast for both scintillators is shown in Fig. 4e. The dashed line at 0.02 denotes the noise level in the inner region, where the lines are no longer resolvable; this value is about 10% of the contrast at the very outer regions of the Siemens star (0.2).

## Results and discussion

The optical characterization of the setup shows a resolution limited by the effective pixel size as apparent in Fig.1a. Lines below 2 ${\upmu }$m period can be still resolved by the setup without the image intensifier. In Fig. 1b a resolution and contrast loss is observed due to the image intensifier as the MCP array constrains the sampling through the cell size and introduces an additional blur broadening the PSF. Using event mode acquisition with centroiding shows that the resolution, but most remarkably the contrast, could be increased. However, the overall resolution is not better than in the image without the intensifier since the period of the MCP (larger than the pixel size) is still limiting the sampling. In fact, the hexagonal structure can be well resolved by centroiding and $10\times$ subsampling (Fig.2e) down to structures as low as 0.4 ${\upmu }$m FWHM—several times smaller than the effective pixel size. However, the sub-pixel reconstruction does not benefit neutron image acquisition in this configuration, as the sampling of the MPC with a hexagonal effective period of 1.8 ${\upmu }$m is the physical limit. This appears consistent with the visually perceived resolution limit in Fig.2c at $\approx$ 2 ${\upmu }$m.

In the radiography experiments at the BNC neutron source, the system can resolve single neutron absorption events. In the case of GOS 5 ${\upmu }$m and GOS 10 ${\upmu }$m distinct clusters with a broad size range are detected. The clusters of GOS 10 ${\upmu }$m are significantly more intense but only somewhat larger as shown by the histogram data in Fig. 3a, and the cluster intensity distribution in Fig. 3e–f. This distribution seems plausible, as two aspects in particular explain this behavior. Firstly, the thicker scintillator offers more interaction volume with the secondary particles ejected from the Gd(n, $\gamma$) reaction and is likely to produce more light. Secondly, a thicker layer causes more light scattering in the granular structure and consequently causes a wider diffusion broadening the average cluster size. However, this also means that light can be expected to travel further away from the point of origin due to multiple scattering and reflection from the substrate, which can no longer be meaningfully assigned to specific clusters. Interestingly, halving the thickness from 10 to 5 ${\upmu }$m, the FWHM of the clusters decreases only by 20%. Hence, using even thinner powdered GOS scintillators might not significantly improve the performance as their neutron absorption and light conversion efficiency will drop disproportionally with respect to the relatively modest benefit in the narrowing of the PSF.

The main contribution to the widening of the light distribution is ascribed to the scattering of light at the grain boundaries within the scintillator. In big clusters, some spots appear up to 15 ${\upmu }$m away from the cluster centers. This range cannot be explained by the range of secondary electrons alone (who have a mean free path <5 ${\upmu }$m). Also, the distribution of the light emission tends to be centrosymmetric, which suggests an isotropic light emission in a small volume in the center and subsequent scattering around it before the photons exit the scintillator surface. Consequently, the shape of the clusters is highly variable and not reproducible. Not only does the image formed on the screen depend on the local variation of the absorbed neutrons (due to grainy structures of GOS) but also on the trajectories of the ejected secondary particles, the light emission (correlated with spatial density variations of the powder) and finally on the scattered photons, of which only a small fraction enters the optical light path of the lens. This leads to the grainy images typical of powder-based scintillators. A pathway to improve this might be the development of structured scintillators where periodic cavities produced by microfabrication methods are filled with the powder. Various such approaches designed to reduce light scattering or improve light guiding properties, mostly evaluated for X-ray imaging applications, have shown some resolution increase with structure sizes of several tens of micrometers^24,25^.

In contrast to GOS scintillators, the GGG scintillator produces mainly isolated spots of comparable size (3–4 pixels) with little indication of cluster formation. Only rarely are several spots detected in close proximity to each other forming small cluster-like structures (two examples in magnified insets in Fig. 3d). It should be mentioned here that it is unclear whether these small clusters come from multiple photons generated in the GGG scintillator. The amplified signal at the output of the image intensifier also originates from a cascade of electrons hitting a fluorescence screen consisting of GOS (P43 phosphor). Hence, the occasionally occurring cluster-like structures could possibly originate from light scattering or reflections in the phosphor of the intensifier. Since the efficiency of the intensifier’s photocathode is $\approx$ 0.15 (545 nm), neutron absorption events yielding, for example, 10 photons, as collected by the objective, would only generate 1–2 primary electrons for amplification. In addition, there is further loss due to the limited collection efficiency of the MCP ($\approx 0.8$). Hence, it is difficult to assess with our acquired data how light-efficient the system is when the GGG scintillator is adopted. In fact, in our case, a high fraction of neutron absorption events might remain completely undetected. To increase the efficiency of event mode imaging with this type of scintillator, one could implement immersion optics with an optical medium between the scintillator and lens. This would also address the efficiency loss due to total reflection in the GGG crystal mentioned before.

The neutron radiographs of the Siemens star show comparable performance of both the GOS 5 ${\upmu }$m and the GGG scintillator with a slight difference in transmission values (GGG 4% higher overall transmission). There is also a slight directional difference in resolution (well recognizable in the contrast variation in Fig. 4d). Possible explanations could be minor errors in drift correction or sampling effects with the MCP. When lines of the Siemens star pattern are parallel to one direction of the hexagonal grid of the MCP the contrast slightly increases in certain ranges at lower periods. Based on the contrast data (Fig. 4e) and visual perception the resolution limit of the system is estimated at 4.0 ${\upmu }$m with the GOS 5 ${\upmu }$m and 4.5 ${\upmu }$m with the GGG scintillator. The resolution is essentially limited by noise and is a factor of $\approx$ 2 lower than the measured optical resolution at comparable magnification. It is worth noting that the contrast of the Siemens star pattern for neutron radiography is only 0.2 in the outer parts of the FoV due to the limited thickness of the Gd absorption layer while it is >0.9 for visible light. This lower contrast together with the small pixel size, strong beam collimation, and thermal setup instabilities make it particularly difficult and time-consuming to resolve structures in the range of 5 ${\upmu }$m.

## Conclusions and outlook

We developed a versatile, modular imaging system for neutron radiography with an adaptable FoV and magnification based on commercially available components and characterized its optical performance focusing on the effects introduced by the image intensifier. Using such photomultiplier optics can effectively amplify the signal especially when scintillators with low light conversion efficiency are used. However, two main limiting factors have to be considered when implementing high-resolution systems with such devices. To reach a pixel size in the range of 1–2 ${\upmu }$m, magnifying optics have to be used before the image intensifier, as its MCP arrays that are limiting the optical resolution have significantly larger periods. Furthermore, the optical magnification should be adjustable to reduce the influence of Moiré artifacts between the MCP and the pixel size of the image sensor or the virtual pixel size in event mode reconstruction. A second limitation is the relatively low quantum efficiency of image intensifiers. Hence, if a low amount of photons are generated during the conversion process in the scintillator and only a fraction of them are collected by the objective lens, some neutron absorption events might be completely missed. Hence, high-NA optics are crucial, especially for crystalline scintillators like GGG immersion objectives coupled with optical medium might be a path worth exploring to reduce the loss due to total reflection in the crystal.

With powder-based GOS scintillators a much higher intensity is observed, however, a grainy image overlay is formed with the transmitted intensity. This is a result of the granular structure of the powder, causing light clusters that can span more than 15 ${\upmu }$m and tend to be larger as the scintillator thickness is increased. The data presented here suggests that the major loss of resolution with GOS scintillators is attributed to photon scattering in the grainy scintillator screen rather than the range of ICEs generated by Gd(n, $\gamma$) as they deposit their energy in the phosphor. Since the process is highly random and not reproducible from cluster to cluster, the CoMs determination becomes more challenging and less reliable. To reduce light scattering, microstructured scintillators with light-guiding properties might be a pathway to improve their resolution. Eventually, particle simulations must be used to calculate the various contributions to cluster formation and deduce key decisive scintillator parameters and physical limits. The first such study with respective experimental verification from event-based imaging systems has recently been performed for another scintillator system^26^.

One big limitation of the presented approach is the high data rate required to be saved and processed, as the vast majority of the pixels do not carry any useful information. Live processing is also unfavorable, as the optimal parameters are not always known a priori. To solve this, the CMOS camera can be replaced by dedicated event-based vision sensors which have already been successfully used with neutrons^21^. They can provide a better temporal resolution and will facilitate more precise neutron localization. Among them, there are also low-budget event cameras with pixel sizes comparable to the used CMOS camera that have been used for similar systems, *e.g.*, event-based single-molecule localization microscopy^27^.

Our results show that both GGG and GOS scintillators reach comparable resolutions in event mode acquisition, however, their light emission characteristics are very different. Consequently, event-based acquisition optics and reconstruction algorithms have to be tailored to each scintillator for optimal performance. While event mode imaging with sub-pixel reconstruction has shown decisive advantages in terms of resolution and contrast for other scintillators at lower resolution^21^, it remains challenging to leverage its advantages in high-resolution systems. However, an event-based scintillator characterization is an invaluable tool for future scintillator development. The influence of thickness, material composition, doping, reflectivity of the substrate, potential micro-structuring of the scintillator, and other parameters can be studied in much more detail, and on an event-basis at microscopic resolution.

## Supplementary Information

Supplementary Information.

## Data Availability

The datasets used and/or analysed during the current study available from the corresponding author on reasonable request.
