# Supplementary material for: Event-based high-resolution neutron image formation analysis using intensified CMOS cameras
Source: Sci Rep. 2024 Nov 6;14:26941. doi: 10.1038/s41598-024-78104-z (PMC11541962; doi:10.1038/s41598-024-78104-z)
Supplement: Supplementary file 1 — Supplementary Information. [file 41598_2024_78104_MOESM1_ESM.pdf]

Supplementary information: Event-based high-resolution Neutron Image Formation Analysis using intensified CMOS cameras

Alex Gustschin[1,*], Yiyong Han[1], Adrian Losko[1], Alexander Wolfertz[1], Daniel S. Hussey[2], László Szentmiklósi[3], Zoltán Kis[3], Pavel Trtik[4], Pierre Boillat[4], Anders Kaestner[4], Markus Strobl[4], Alessandro Tengattini[5,6], Lukas Helfen[5], and Michael Schulz[1]

[1]Technical University of Munich, Heinz Maier-Leibnitz Zentrum (MLZ), Lichtenbergstr. 1, 85748, Garching, Germany
[2]National Institute of Standards and Technology (NIST), Gaithersburg, MD 20899-8461, USA
[3]Nuclear Analysis and Radiography Department, Centre for Energy Research, Budapest, Hungary
[4]Laboratory for Neutron Scattering and Imaging, Paul Scherrer Institut (PSI), 5232 Villigen, Switzerland
[5]Institut Laue-Langevin (ILL), 38042 Grenoble Cedex 9, France
[6]Univ. Grenoble Alpes, Grenoble INP, CNRS, 3SR, F-38000 Grenoble, France
[*]alex.gustschin@frm2.tum.de

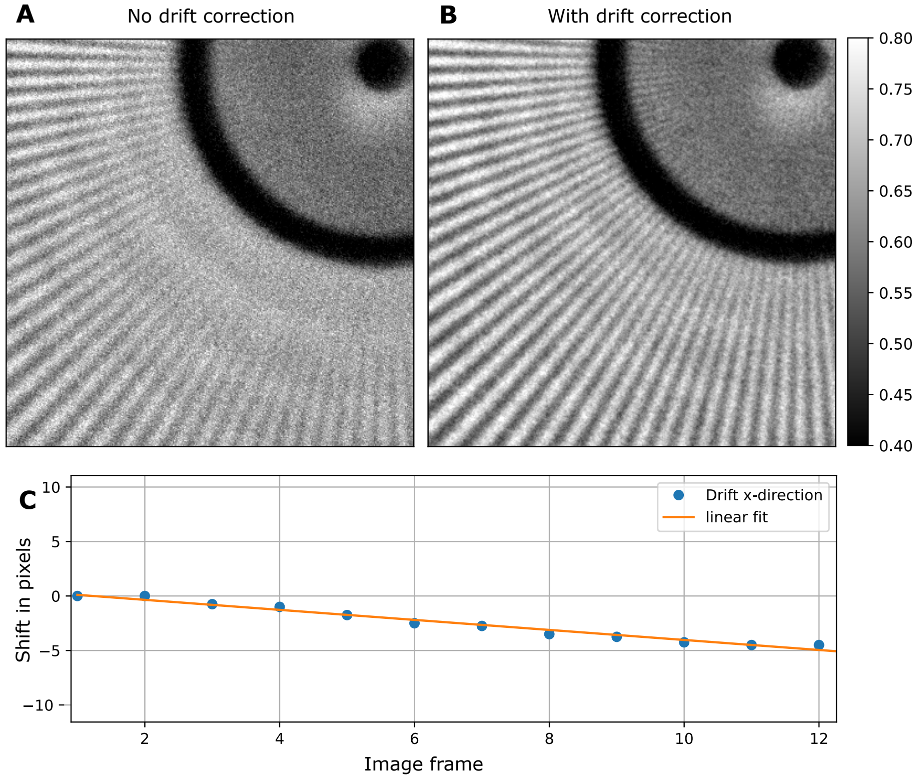

Figure S1. (A) Image without drift correction by adding consecutive temporal frames. (B) Image with drift correction using the digital image correlation method described in the main manuscript. (C) Exemplary shifts in pixels were determined for consecutive image frames in x-direction (blue dots), and linear fit used to compensate for the sample drift in the corrected image. The sample drift of $\approx 5\mu m$ in x-direction over 6h total sample exposure caused an obvious resolution loss in the vertical lines as seen in (A).
